# Composite Proton Exchange Membranes with Interlayer Structure Containing Functional Catalyst Particles for Water Electrolysis

**DOI:** 10.1021/acsami.5c08461

**Published:** 2025-09-18

**Authors:** Zheyu Zhang, Masis Sirim, Dominika Baster, Mario El Kazzi, Andrea Testino, Lorenz Gubler

**Affiliations:** † 28498 PSI Center for Energy and Environmental Sciences, Villigen PSI 5232, , Switzerland; ‡ STI SMX-GE, École Polytechnique Fédérale de Lausanne, Lausanne 1015, Switzerland

**Keywords:** PEM water electrolysis, membrane, cerium-zirconium oxide, radical scavenger, Pt, gas recombination, interlayer

## Abstract

The development of more cost-effective and efficient proton exchange membrane (PEM) water electrolysis cells requires the use of thinner membranes with extended lifetimes and reduced gas crossover. One approach to address these challenges involves the incorporation of radical scavengers to mitigate radical-induced ionomer degradation and gas recombination catalysts to promote the conversion of crossover hydrogen and oxygen to water. The positioning effects of these two catalyst interlayers in composite membranes were investigated. Results reveal that placing a Ce_0.5_Zr_0.5_O_2_ radical scavenger interlayer near the cathode notably reduces the ionomer degradation rate, compared to its placement near the anode. The Ce content in cerium-zirconium oxide was optimized, with Ce_0.25_Zr_0.75_O_2_ demonstrating the highest radical scavenging activity. The Pt gas recombination interlayer is confirmed to be more effective when positioned near the anode. This Pt interlayer, however, was found to induce additional ionomer degradation and was replaced by a bi-functional catalyst interlayer of Pt/Ce_0.25_Zr_0.75_O_2_. Consequently, the composite membrane with a Ce_0.25_Zr_0.75_O_2_ interlayer near the cathode and a Pt/Ce_0.25_Zr_0.75_O_2_ interlayer near the anode yields the lowest rates of both ionomer degradation and hydrogen crossover, demonstrating a projected membrane lifetime 7.4 times longer and a H_2_ in O_2_% 4.4 times lower compared to the blank membrane.

## Graphical abstract

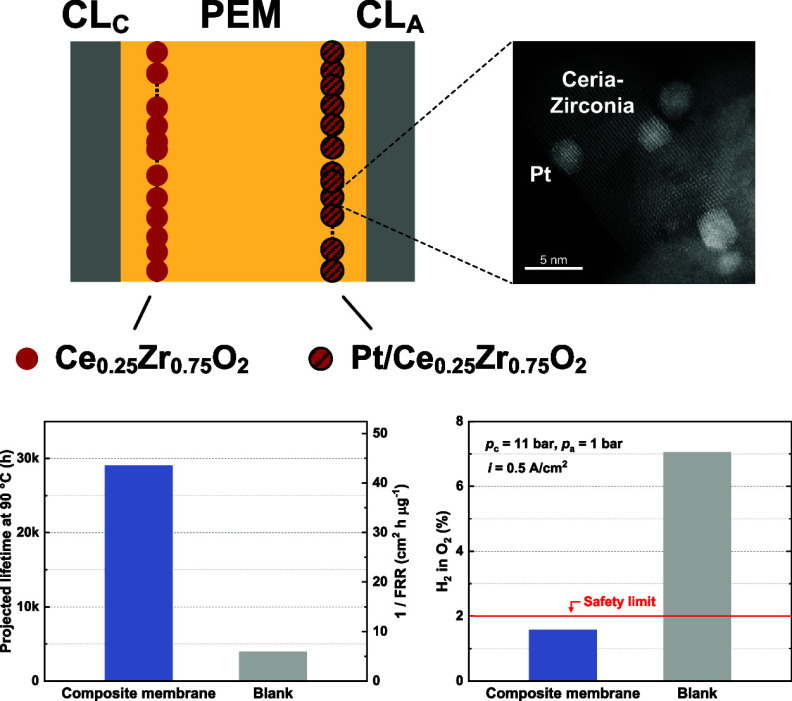

## 1 Introduction

Proton exchange membrane (PEM) water electrolysis is an anticipated core technology to produce hydrogen from renewable energy sources for power-to-X processes.^1^ Performance, cost and durability are widely considered as the three main challenges to the advancement of this technology.^2,3^ Enhancing the durability of PEM water electrolysis cells is crucial for reducing system costs and improving the economic competitiveness of the produced hydrogen.^4–6^ In the literature, stack lifetimes ranging from 60,000 to 100,000 h have been reported,^7^ with projections anticipating an increase to over 125,000 h by 2050.^8^ Among the factors influencing cell durability, chemical degradation of the membrane plays an important role,^9^ and is of particular relevance when thin membranes of ∼50 μm thickness or less are employed.

The irreversible chemical membrane degradation is typically driven by reactive oxygen species (ROS) that are generated on catalytic surfaces due to gas crossover.^5^ These ROS include hydrogen peroxide (H_2_O_2_) and its associated radicals, such as hydroxyl (HO^•^) and hydroperoxyl (HOO^•^) radicals.^5^ The radicals attack the polymer structure, leading to chain scission, and loss of ion exchange groups and other structural constituents, resulting in membrane thinning and reduced ionic conductivity.^10^ In the case of perfluoroalkylsulfonic acid (PFSA)-based membranes, such as Nafion, the release of fluoride is detected in the cell effluent water, serving as an indicator of polymer degradation.^10,11^

It is generally accepted that this degradation primarily occurs near the cathode side, although a comprehensive understanding remains to be elucidated.^5,12–18^ Chandesris et al. developed a one-dimensional model addressing the degradation mechanism, involving the formation of H_2_O_2_ at the cathode as a result of oxygen crossover, followed by the generation of radicals via Fenton reactions.^14^ Experimental measurements of the fluoride release rate (FRR) supported the model, demonstrating higher FRR values at the cathode compared to the anode.^14^ This conclusion aligns with the observations made by Fouda-Onana et al.^12^ and Frensch et al.^17^ It is thought that H_2_O_2_ is produced both chemically via the reaction of crossover hydrogen and oxygen gases on platinum (Pt) surfaces accompanied by radical formation, and electrochemically through a two-electron pathway at the cathode under low-potential conditions.^19^ H_2_O_2_ possesses a diffusion length on the order of millimeters to centimeters, allowing it to traverse the membrane.^20^ During its diffusion, H_2_O_2_ can decompose to form radicals, either uncatalyzed or catalyzed by certain metal ions.^21^

An effective strategy for mitigating membrane chemical degradation is to incorporate a radical scavenger, such as a cerium (Ce)-based catalyst.^16,19,22,23^ It reacts with ROS while maintaining a dynamic equilibrium of the Ce^4+^/Ce^3+^ redox couple.^20^ Although Ce-based radical scavengers have been extensively studied in PEM fuel cell research,^24–28^ their application in PEM water electrolysis remains relatively limited to date. Current reports mainly describe the homogeneous incorporation of the catalyst inside the membrane.^29–32^ However, since the membrane degradation predominantly takes place near the cathode, such a homogeneous distribution may not represent the most efficient or optimal placement of the radical scavenger. We hypothesize that positioning the Ce-based radical scavenger as an interlayer near the cathode side would be a rational approach, as this placement is expected to ensure effectiveness of the radical scavenger by likely targeting the region close to the highest ROS concentration. The impact of radical scavenger positioning on mitigating membrane degradation has been largely overlooked in the literature so far, and our study aims to shed some light on this issue.

Furthermore, another obstacle in the development of PEM water electrolysis cells with thinner membranes is the increased hydrogen crossover. Recombination catalysts, such as Pt, have been employed to reduce the rate of crossover hydrogen reaching the anode side.^33^ Klose et al. proposed an ideal placement of the recombination catalyst layer within the membrane, taking into account the partial pressures and effective diffusion coefficients of hydrogen and oxygen in water under a set of given conditions.^34^ Specifically, in a typical PEM water electrolysis cell operating at 80 °C—where the cathode H_2_ chamber is usually pressurized while the anode O_2_ chamber is maintained at ambient pressure—the optimal location for the recombination layer is near the anode side. This interlayer membrane configuration was adopted by Stähler et al.^35^ and Abbas et al.^36^ Brundiers et al. developed an analytical model explaining the effect of the recombination layer at a cell level.^37^ Nevertheless, as reported in our previous studies, incorporating Pt as a recombination catalyst was found to exacerbate ionomer degradation, likely due to its role in catalyzing additional radical formation.^29,38^ A Pt/Ce_*x*_Zr_1‑*x*_O_2_ bi-functional catalyst was instead proposed as a replacement for Pt, seeking to concurrently mitigate hydrogen crossover and ionomer degradation.^38^ Building on the novel application of this bi-functional catalyst and informed by the optimal placement suggested in the literature, we recommend positioning a Pt/Ce_*x*_Zr_1*‑x*_O_2_ interlayer near the anode side of the membrane, representing an unexplored approach in addressing the problem.

In this report, we began by investigating the effective positioning of a model radical scavenger, Ce_0.5_Zr_0.5_O_2_, placing a spray-coated Ce_0.5_Zr_0.5_O_2_ interlayer near either the anode or cathode within the membrane. The FRRs of the two composite membranes were monitored and compared over 100 h of constant current measurements at 2 A/cm^2^. The Ce content in Ce_*x*_Zr_1*‑x*_O_2_ was further optimized for the highest radical scavenging capability using an ex situ Fenton test, identifying Ce_0.25_Zr_0.75_O_2_ as the optimal composition. This material was therefore placed near the cathode side, which had been determined as the more effective location for radical scavenger within the composite membrane. The positioning of a gas recombination catalyst interlayer, with Pt as a model material, was additionally evaluated for its role in reducing hydrogen crossover. Consistent with the literature, the Pt interlayer was found to be more effective near the anode under the experimental conditions. To address the additional ionomer degradation induced by the Pt interlayer, a bi-functional catalyst Pt/Ce_0.25_Zr_0.75_O_2_ interlayer was introduced as a substitute. Building on these findings, a composite membrane configuration featuring Ce_0.25_Zr_0.75_O_2_ near the cathode and Pt/Ce_0.25_Zr_0.75_O_2_ near the anode was developed and examined, achieving concurrently an enhanced mitigation of ionomer degradation and hydrogen crossover.

## 2 Results and Discussion

### 2.1 Effective Positioning of Ce_0.5_Zr_0.5_O_2_ Radical Scavenging Interlayer

Two configurations of composite membranes were prepared, each incorporating a Ce_0.5_Zr_0.5_O_2_ interlayer positioned either near the cathode (*Ce*_0.5_ *Zr*_0.5_ *O*_2_ *(ca)*) or near the anode (*Ce*_0.5_ *Zr*_0.5_ *O*_2_ *(an)*) of a Nafion NR 212 membrane, as illustrated in Figure 1a,b. An ionomer layer was deposited over the Ce_0.5_Zr_0.5_O_2_ interlayer to provide electrical insulation from the catalyst layer. Ideally, this ionomer layer should be sufficiently thin to minimize additional ohmic resistance, yet thick enough to ensure effective insulation during long-term cell operation. The optimal thickness of the ionomer layer remains to be determined through future investigations. The SEM cross-sectional image (Figure 1c), using *Ce*_0.5_ *Zr*_0.5_ *O*_2_ *(ca)* as an example, shows a well-defined composite membrane structure, comprising a 47.6 ± 0.6 μm thick Nafion NR 212 membrane, a 7 ± 1 μm thick Ce_0.5_Zr_0.5_O_2_ interlayer and a 5 ± 1 μm thick ionomer layer.

**Figure 1:**
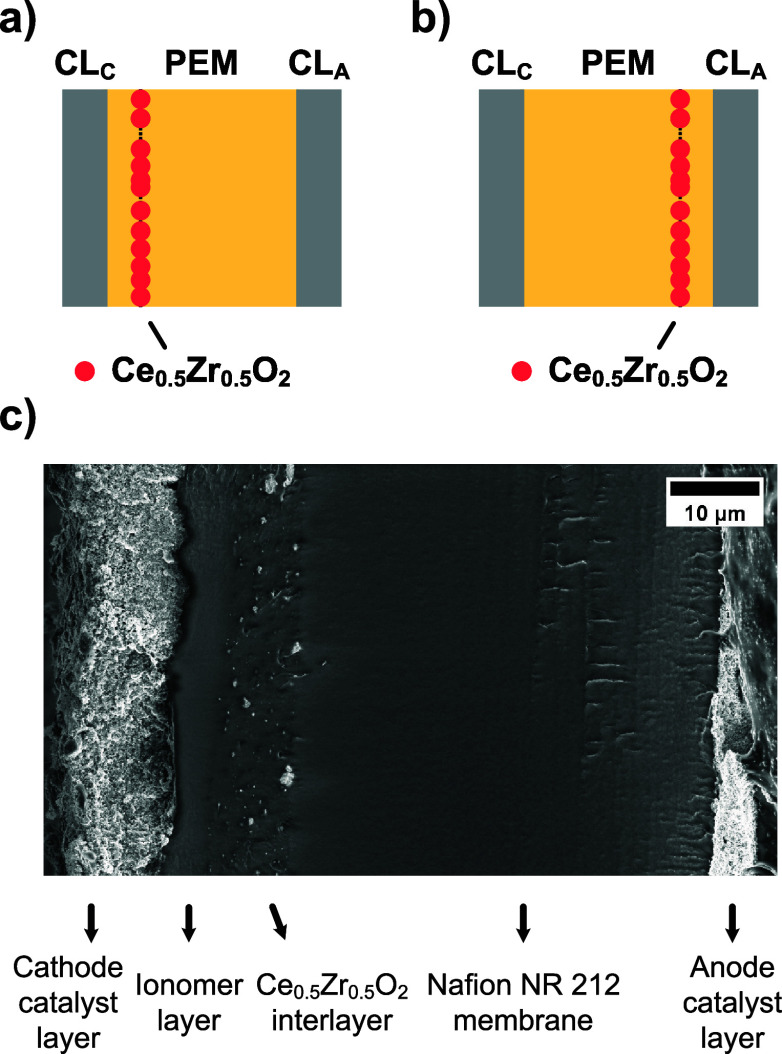
Schematic representation of catalyst coated membranes (CCMs) containing the composite membranes of *Ce*_0.5_ *Zr*_0.5_ *O*_2_ *(ca)* (a) and *Ce*_0.5_ *Zr*_0.5_ *O*_2_ *(an)* (b) (PEM = proton exchange membrane, CL_c_ = cathode catalyst layer, CL_a_ = anode catalyst layer), and cross-sectional SEM image of a CCM containing the composite membrane of *Ce*_0.5_ *Zr*_0.5_ *O*_2_ *(ca)* (c).

The radical scavenging effectiveness of the Ce_0.5_Zr_0.5_O_2_ interlayer was evaluated in 100-h constant current measurements at 2 A/cm^2^, conducted at 90 °C under differential pressures (*p*_c_ = 11 bar and *p*_a_ = 1 bar). The fluoride release rates, calculated based on electro-osmotic drag water rates (Table S1) and fluoride concentrations in the cathode effluent water, are shown in Figure 2a. Blank measurements were performed using three membranes without any Ce_0.5_Zr_0.5_O_2_ interlayers (individual results presented in Figure S1). Their averaged FRR, 0.17 ± 0.01 (μg·cm^–2^·h^–1^), is represented by a solid horizontal line, with the error bar indicated by a grey-shaded region.

**Figure 2:**
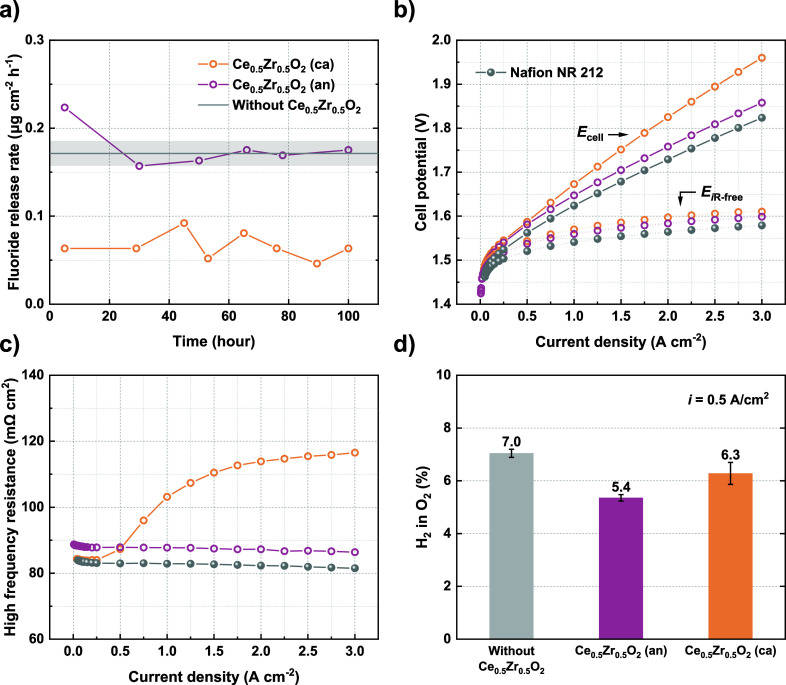
Fluoride release rates determined during 100-h constant current tests (a), polarization curves and *iR*-free potentials (b), the corresponding high-frequency resistances (c), and the measured H_2_ in O_2_% at 0.5 A/cm^2^ (d) for different membrane configurations.

The results demonstrate that positioning the Ce_0.5_Zr_0.5_O_2_ interlayer near the cathode effectively reduces the FRR compared to its placement near the anode — the latter exhibits an FRR similar to the blank level. The average FRR over the 100 h for *Ce*_0.5_ *Zr*_0.5_ *O*_2_ *(an)* is 0.18 ± 0.02 (μg·cm^–2^·h^–1^), whereas for *Ce*_0.5_ *Zr*_0.5_ *O*_2_ *(ca)* it is 0.07 ± 0.01 (μg·cm^–2^·h^–1^), corresponding to a reduction of 61% ± 7%. This finding is in line with literature analysis, where the cathode is identified as the primary location of ROS generation responsible for membrane degradation.^12,14^ Placing the radical scavenger closer to this region is beneficial in lowering the ROS concentrations, thereby improving the chemical stability of the membrane. Reactions involving ROS with the Ce^4+^/Ce^3+^ redox couple in cerium-zirconium oxide commonly include:^39^

1 $${\mathrm{HO}}^{•}+{\mathrm{Ce}}^{3+}+H^{+}\to {\mathrm{Ce}}^{4+}+H_{2}O$$

2 $${\mathrm{HOO}}^{•}+{\mathrm{Ce}}^{3+}+H^{+}\to {\mathrm{Ce}}^{4+}+H_{2}O_{2}$$

3 $$H_{2}O_{2}+{\mathrm{Ce}}^{4+}\to {\mathrm{Ce}}^{3+}+{\mathrm{HOO}}^{•}+H^{+}$$

4 $${\mathrm{HOO}}^{•}+{\mathrm{Ce}}^{4+}\to {\mathrm{Ce}}^{3+}+O_{2}+H^{+}$$

The oxidative strength of these ROS follows the order: HO^•^ > H^•^ > HOO^•^ > H_2_O_2_.^19^ Notably, as HO^•^ has a short diffusion length of ∼40 nm in PFSA membranes,^20^ direct scavenging of HO^•^ produced in the cathode catalyst layer by Ce_0.5_Zr_0.5_O_2_ is unlikely due to the presence of an ionomer layer with a thickness in the range of 5 μm. Nevertheless, the relatively stable ROS, H_2_O_2_, and HOO^•^, can diffuse from the cathode into the membrane toward the anode.^20^ Depending on the position of the Ce_0.5_Zr_0.5_O_2_ interlayer, they can be consumed by Ce^4+^ along their diffusion pathway, particularly for HOO^•^ whose diffusion length is ∼20 μm, shorter than the distance to the Ce_0.5_Zr_0.5_O_2_ interlayer when positioned near the anode.^20^ Scavenging these species closer to the cathode in a timely manner can, therefore, reduce the extent of membrane degradation. Furthermore, H_2_O_2_ and HOO^•^ participate in reactions that lead to the formation of HO^•^, such as the Fenton reaction involving H_2_O_2_, as follows:^19^

5 $$H_{2}O_{2}+H^{+}+{\mathrm{Fe}}^{2+}\to {\mathrm{Fe}}^{3+}+{\mathrm{HO}}^{•}+H_{2}O$$

Here, iron ions could be present from feedwater impurities. The decomposition of H_2_O_2_ can also be catalyzed by other multivalent metal ions, including Cu^2+^ and Ti^3+^.^19^ These ions may arise from the corrosion of system tubing or cell components.^19,21^ For example, Rakousky et al. identified titanium migration to the anode after 1,150 h of cell operation,^40^ while Yu et al. reported Pt dissolution in the cathode.^41^ A reduced H_2_O_2_ concentration near the cathode side, achieved using Ce_0.5_Zr_0.5_O_2_ in the configuration of *Ce*_0.5_ *Zr*_0.5_ *O*_2_ *(ca)*, mitigates subsequent HO^•^ formation as H_2_O_2_ diffuses through the membrane and interacts with metal ion impurities. Overall, these combined effects in mitigation of ROS concentrations account for the observed lower FRR when the Ce_0.5_Zr_0.5_O_2_ interlayer is positioned near the cathode, as opposed to be positioned near the anode.

Polarization curves (Figure 2b) indicate a generally higher cell potential for *Ce*_0.5_ *Zr*_0.5_ *O*_2_ *(ca)* than *Ce*_0.5_ *Zr*_0.5_ *O*_2_ *(an)*, mostly due to increased high-frequency resistance (HFR) (Figure 2c). The *iR*-free cell potentials for both configurations are similar, as expected. At 2 A/cm^2^, the measured HFR for *Ce*_0.5_ *Zr*_0.5_ *O*_2_ *(an)* is 87 mΩ·cm^2^, whereas for *Ce*_0.5_ *Zr*_0.5_ *O*_2_ *(ca)*, it is 114 mΩ·cm^2^ (Figure 2c). It is worth noting that the HFR varies significantly with current density in the case of *Ce*_0.5_ *Zr*_0.5_ *O*_2_ *(ca)*, starting to increase at 0.25 A/cm^2^ from 84 mΩ·cm^2^ to 117 mΩ·cm^2^ at 3 A/cm^2^. This may be attributed to interfacial effects resulting from an additional resistance at the interface of the membrane layers, as also observed by Klose et al.^34^ However, the absence of such an effect for *Ce*_0.5_ *Zr*_0.5_ *O*_2_ *(an)* is interesting. Further investigation (Figure S2) using composite membranes containing only an ionomer layer, without any Ce_0.5_Zr_0.5_O_2_ interlayers, confirms that the interfacial effects on HFR is only observed when the ionomer layer, and consequently the interface, is located near the cathode, instead of the anode. This may be related to an inhomogeneous distribution of mechanical stress across the membrane, as the anode is supported by a rigid Ti-felt whereas the cathode is supported by a relatively soft carbon non-woven, making a delamination more likely to occur near the cathode side. The differential pressure setting of 11 bar at the cathode and ambient at the anode could further exacerbate this situation. As the current density increases, the rate of electro-osmotic water drag increases proportionally, potentially causing water to accumulate inside the delaminated interface and thereby increasing the ohmic resistance. It is believed that this water can be re-absorbed into the membrane as conditions change, since the increased HFR was found to be reversible. At current densities below 0.25 A/cm^2^, the HFRs for *Ce*_0.5_ *Zr*_0.5_ *O*_2_ *(an)* and *Ce*_0.5_ *Zr*_0.5_ *O*_2_ *(ca)* are comparable, falling within the CCM-to-CCM variations. Notably, the HFR for *Ce*_0.5_ *Zr*_0.5_ *O*_2_ *(ca)* overlaps mostly with that for the Nafion NR 212 reference within this current density range.

The measured H_2_ in O_2_% is the highest for membranes without a Ce_0.5_Zr_0.5_O_2_ interlayer, reaching 7.0% at 0.5 A/cm^2^ (Figure 2d). This value decreases to 6.3% for *Ce*_0.5_ *Zr*_0.5_ *O*_2_ *(ca)*, and is the lowest for *Ce*_0.5_ *Zr*_0.5_ *O*_2_ *(an)*, at 5.4%. The incorporation of the Ce_0.5_Zr_0.5_O_2_ interlayer may contribute to this reduction via the following effects: (I) The addition of the interlayer slightly increases the composite membrane thickness, extending the hydrogen diffusion length and therefore marginally reducing the rate of hydrogen crossover. (II) The interlayer contains 50 wt % Ce_0.5_Zr_0.5_O_2_ particles and 50 wt % ionomer. The reduced ionomer content within the interlayer limits the available water channels for the dissolved hydrogen to diffuse through, thereby decreasing the hydrogen crossover. (III) Ce_0.5_Zr_0.5_O_2_ particles may exhibit weak catalytic activity for the recombination of crossover hydrogen and oxygen under the current conditions, providing adsorption surfaces that facilitate the reaction.^42,43^ Given that the reactant ratio near the anode is closer to the ideal reaction stoichiometry of 2:1 for hydrogen and oxygen (as will be elaborated in a later section), this leads to a lower H_2_ in O_2_% for *Ce*_0.5_ *Zr*_0.5_ *O*_2_ *(an)* than *Ce*_0.5_ *Zr*_0.5_ *O*_2_ *(ca)*. On the other hand, considering the experimental error bars in Figure 2d, the differences are only slightly more than the sample variations.

### 2.2 Ex Situ Study for an Optimized Ce Content in Ce_*x*_Zr_1‑*x*_O_2_

While the effectiveness of Ce_0.5_Zr_0.5_O_2_ with respect to its positioning has been demonstrated in the previous section, it is beneficial to further explore the optimal Ce content for radical scavenging in cerium-zirconium oxide, beyond a Ce/Zr ratio of 1. To address this question, powders of Ce_*x*_Zr_1–*x*_O_2_ with three different Ce contents (*x* = 0.25, 0.5, and 0.75) were synthesized. Phase characterization using powder X-ray diffraction (Figure 3a) reveals a peak shift toward lower 2θ angles with increasing Ce content, consistent with increased lattice spacing due to the substitution of Zr^4+^ (0.84 Å) by Ce^4+^ (0.97 Å).^44,45^ A gradual peak broadening is observed as the Ce content increases, which may be attributed to reduced crystallite size and/or increased lattice strain. Nitrogen physisorption measurements of the powders exhibit a type IV isotherm with a H1 type hysteresis for Ce_0.25_Zr_0.75_O_2_ and Ce_0.5_Zr_0.5_O_2_, and a type II isotherm with a H4 type hysteresis for Ce_0.75_Zr_0.25_O_2_ (Figure 3b).^46^ The hysteresis feature for Ce_0.25_Zr_0.75_O_2_ and Ce_0.5_Zr_0.5_O_2_ indicates the presence of uniformly distributed spherical or cylindrical mesopores wider than ∼4 nm.^46,47^ This aligns with the pore size distribution results (Figure S3), which show both micropores (centering at ∼1–2 nm) and mesopores (centering at ∼3–3.5 nm) for the two samples. The cumulative pore volumes are 0.146 cm^3^/g and 0.121 cm^3^/g for Ce_0.25_Zr_0.75_O_2_ and Ce_0.5_Zr_0.5_O_2_, respectively. Mesopores are generally absent in Ce_0.75_Zr_0.25_O_2_, whose cumulative pore volume is significantly lower, being 0.022 cm^3^/g. Its isotherm, with a slight hysteresis feature, indicates the presence of narrow slit-like pores.^46,47^ Among the three compositions, Ce_0.25_Zr_0.75_O_2_ shows the highest BET surface area of 71 m^2^/g. The estimated crystallite sizes for all samples, based on the BET surface areas and by assuming spherical particles, ranges from 13 to 15 nm, consistent with those obtained from Rietveld refinement in the previous work (Table S2).^38^

**Figure 3:**
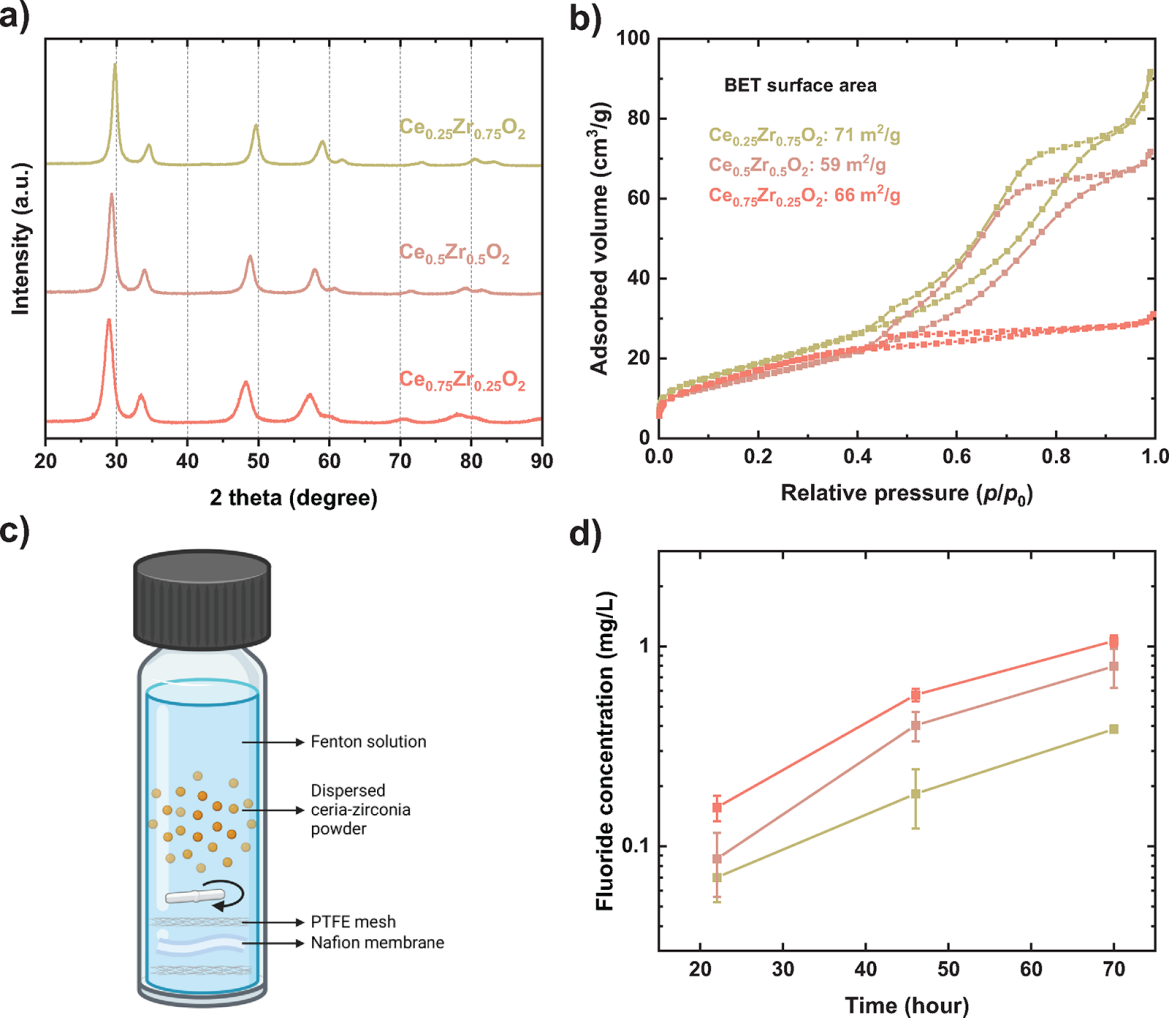
PXRD patterns of the synthesized Ce_*x*_Zr_1–*x*_O_2_ (*x* = 0.25, 0.5, and 0.75) (a), N_2_ physisorption isotherms and surface areas calculated using the BET method (b), schematic illustration of the ex situ Fenton test setup^48^ (c), and fluoride concentrations monitored during the ex situ Fenton tests (d) for these synthesized cerium-zirconium oxides.

To compare the radical scavenging performance of Ce_*x*_Zr_1‑*x*_O_2_ with varying Ce contents, an ex situ Fenton test was carried out. In this setup, each sample powder and a piece of Nafion NR 212 membrane were placed in a vial containing freshly prepared Fenton’s reagent, as illustrated in Figure 3c. The amount of Ce_*x*_Zr_1*‑x*_O_2_ was normalized to the same Ce mass across different compositions to ensure consistency. Fenton’s reagent contains a high concentration of free radicals,^49^ which can either be scavenged by Ce_*x*_Zr_1*‑x*_O_2_, or attack the Nafion membrane resulting in the release of fluoride. Consequently, fluoride concentration in the vial was monitored to assess the extent of the membrane degradation and, in turn, evaluate the radical scavenging activity of Ce_*x*_Zr_1–*x*_O_2_. As shown in Figure 3d, the suspension with Ce_0.25_Zr_0.75_O_2_ yields the lowest fluoride concentration over the 70-h test period, indicating its most effective radical scavenging activity under the specified conditions. Higher Ce contents (*x* = 0.5 and 0.75) sequentially increase the resulting fluoride concentrations, with Ce_0.75_Zr_0.25_O_2_ demonstrating the lowest radical scavenging activity. Literature suggests that Ce^3+^ concentration per Ce atom in cerium-zirconium oxide generally increases with the Zr content.^50–52^ Therefore, in the samples studied, the Ce^3+^/total Ce ratio is expected to increase in the order of: Ce_0.75_Zr_0.25_O_2_ < Ce_0.5_Zr_0.5_O_2_ < Ce_0.25_Zr_0.75_O_2_. With a higher Ce^3+^ concentration, as in Ce_0.25_Zr_0.75_O_2_, more HO^•^ can be effectively scavenged via eq 1, while Ce^3+^ itself is regenerated via eq 3, leading to the observed higher radical scavenging activity. The results of X-ray photoelectron spectroscopy measurements acquired at Ce 3d core level confirmed that Ce_0.25_Zr_0.75_O_2_ exhibited a higher Ce^3+^ concentration compared to Ce_0.5_Zr_0.5_O_2_ (Figure S4 and Tables S3 and S4). However, a similarly high Ce^3+^ concentration was also observed in Ce_0.75_Zr_0.25_O_2_, the cause of which remains unclear at this stage.

### 2.3 Effective Positioning of Pt Gas Recombination Interlayer and Substitution of Pt with Bi-functional Catalyst Pt/Ce_0.25_Zr_0.75_O_2_

Based on the findings from the previous two sections, Ce_0.25_Zr_0.75_O_2_ was positioned as an interlayer near the cathode of the composite membrane. This configuration was maintained in subsequent experiments.

Meanwhile, the high rate of hydrogen crossover, exemplified by the blank membranes in Figure 2d, presents a significant challenge to the safe operation of the electrolysis cell. It is understood that hydrogen crossover occurs mainly through the diffusion of dissolved hydrogen gas in the aqueous domain of the hydrated membrane.^53^ The rate of hydrogen crossover typically increases with increasing cathode pressure and/or decreasing membrane thickness. While elevated cathode pressure is preferred for eliminating or reducing the need of mechanical hydrogen compression, and thin membranes are favored for reducing ohmic losses during cell operation,^2^ one approach to mitigate pronounced hydrogen crossover is to incorporate a gas recombination catalyst, such as Pt.^33^ This catalyst recombines the crossover hydrogen and oxygen to water, as

6 $$2H_{2}+O_{2}\;\overset{\mathrm{Pt}}{⃗}\;2H_{2}O$$

where the ideal reaction stoichiometry for hydrogen and oxygen is 2:1. If the amounts of reactants do not meet the stoichiometric ratio, the reaction will remain incomplete. In cases where oxygen is the limiting reactant, excess hydrogen gas will continue crossing over through the membrane to the anode side, thereby leading to a high hydrogen content in the anode oxygen stream.

The ideal position inside the membrane for a gas recombination interlayer, where the crossover hydrogen and oxygen satisfy the stoichiometric ratio, is^34^

7 $$x={\left(\frac{2K_{{P,O}_{2}}^{\mathrm{diff}}{\;p}_{O_{2}}^{\mathrm{an}}}{K_{{P,H}_{2}}^{\mathrm{diff}}{\;p}_{H_{2}}^{\mathrm{ca}}}+1\right)}^{-1}.\delta_{\mathrm{mem}}$$

Here, $K_{{P,O}_{2}}^{\mathrm{diff}}$ and $K_{{P,H}_{2}}^{\mathrm{diff}}$ are the permeability coefficients of O_2_ and H_2_ in the membrane, while $p_{O_{2}}^{\mathrm{an}}$ and $p_{H_{2}}^{\mathrm{ca}}$ are the partial pressures of O_2_ and H_2_, respectively, *δ*_mem_ is the membrane thickness, and *x* represents the position of the gas recombination interlayer inside the membrane, with its direction pointing from the cathode toward the anode. Under the current testing conditions of 90 °C, 11 bar cathode pressure and ambient anode pressure, an interlayer position close to the anode side, where *x* = 0.98·δ_mem_, is determined based on extrapolation of data from Schalenbach et al.^54^

To validate the effective positioning of the gas recombination interlayer, a Pt interlayer with a loading of 0.04 mg_Pt_/cm^2^ was incorporated near either the anode or the cathode of the composite membrane, as illustrated in Figure 4a,b. An ionomer layer was placed over the Pt interlayer when positioned near the anode to insulate it from the catalyst layer. In the case of the Pt interlayer positioned near the cathode, this anode ionomer layer was also added to the membrane anode side for consistency. The anode H_2_ in O_2_% was measured in a range of current densities from 0.5–3 A/cm^2^, followed by a 100-h constant current measurement at 2 A/cm^2^ with periodic monitoring of fluoride release rate (Figure 4d). As shown in Figure 4e, the configuration *Ce*_0.25_ *Zr*_0.75_ *O*_2_ *(ca) - Pt (an)* indeed exhibits a generally lower H_2_ in O_2_% compared to *Ce*_0.25_ *Zr*_0.75_ *O*_2_ *(ca) - Pt (ca)*. At 0.5 A/cm^2^, a reduction from 5.0% to 2.9% is seen when the Pt interlayer is changed from near the cathode to near the anode side. This observation confirms the influence of the gas recombination interlayer position, considering the yield of the recombination reaction.

**Figure 4:**
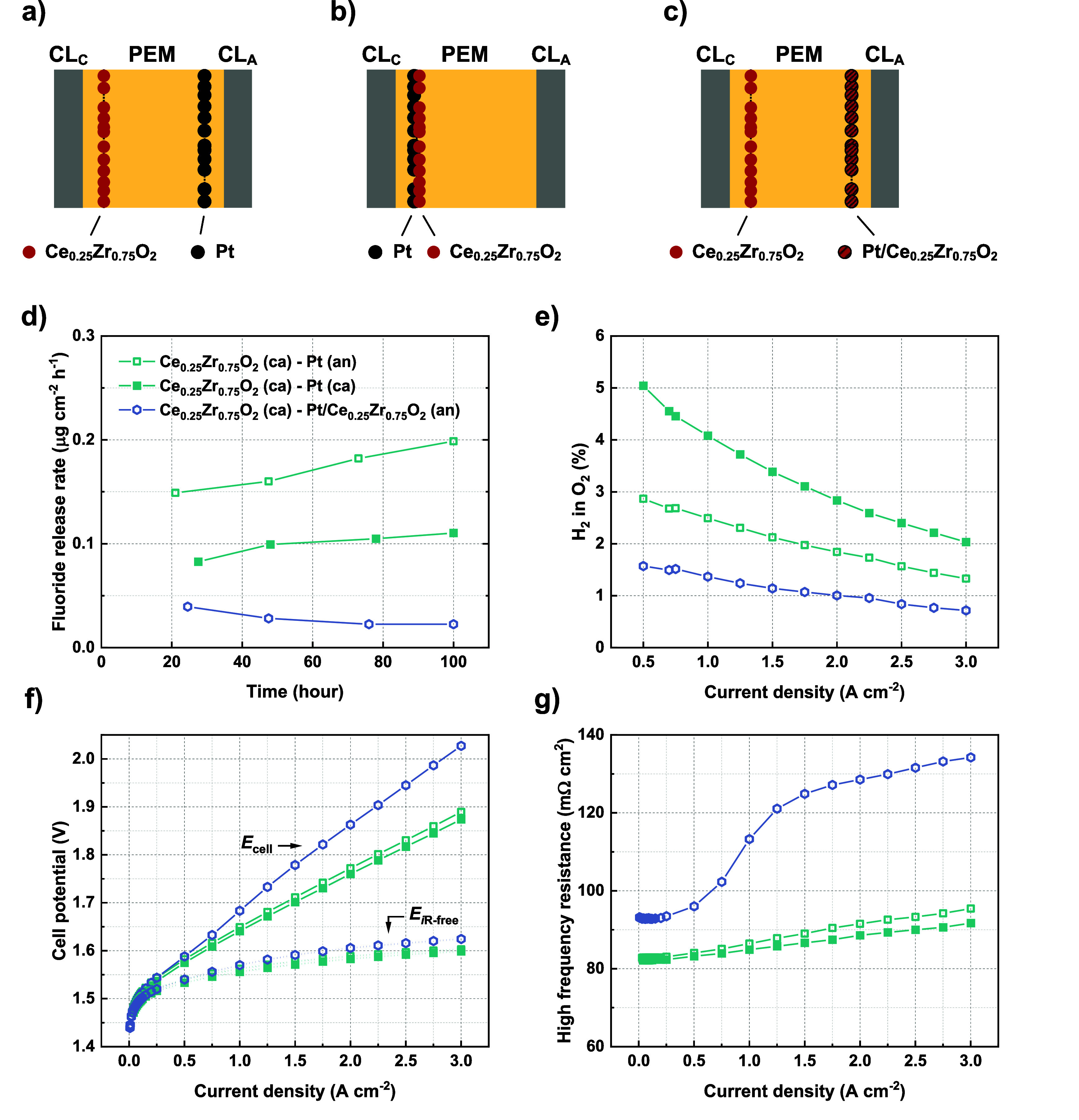
Schematic representation of CCMs containing the composite membranes of *Ce*_0.25_ *Zr*_0.75_ *O*_2_ *(ca) - Pt (an)* (a), *Ce*_0.25_ *Zr*_0.75_ *O*_2_ *(ca) - Pt (ca)* (b), and *Ce*_0.25_ *Zr*_0.75_ *O*_2_ *(ca) - Pt/Ce*_0.25_ *Zr*_0.75_ *O*_2_ *(an)* (c) (PEM = proton exchange membrane, CL_c_ = cathode catalyst layer, CL_a_ = anode catalyst layer), fluoride release rates determined during 100-h constant current tests (d), measured H_2_ in O_2_% in the current density range of 0.5–3 A/cm^2^ (e), polarization curves and *i*R-free potentials (f), and the corresponding high-frequency resistances (g) for these composite membranes.

On the other hand, a higher fluoride release rate was determined for *Ce*_0.25_ *Zr*_0.75_ *O*_2_ *(ca) - Pt (an)* than for *Ce*_0.25_ *Zr*_0.75_ *O*_2_ *(ca) - Pt (ca)* (Figure 4d), indicating an increased rate of membrane degradation when the Pt interlayer is placed near the anode. Since Pt is known to catalyze not only the gas recombination of hydrogen and oxygen, but also radical formation,^29^ a higher radical concentration is likely to induce more rapid membrane degradation when these radicals are generated far from the radical scavenger and cannot be timely scavenged, as in the case of *Ce*_0.25_ *Zr*_0.75_ *O*_2_ *(ca) - Pt (an)*. In contrast, for *Ce*_0.25_ *Zr*_0.75_ *O*_2_ *(ca) - Pt (ca)*, since the radical scavenger is positioned close to the Pt, the produced radicals can be scavenged effectively by Ce_0.25_Zr_0.75_O_2_, resulting in a lower membrane degradation rate.

The dilemma in the use of Pt with reduced hydrogen crossover but promoted radical formation can be addressed by employing a bi-functional catalyst Pt/Ce_*x*_Zr_1*‑x*_O_2_, as proposed in our previous study.^38^ The substitution of Pt with Pt/Ce_*x*_Zr_1*‑x*_O_2_ was demonstrated to concurrently mitigate hydrogen crossover and ionomer degradation. Here, we adopted a similar approach. Specifically, Pt/Ce_0.25_Zr_0.75_O_2_ was synthesized via a one-pot polyol method, with calcination carried out in a reducing atmosphere. Nano-sized Pt metal particles can be directly formed on the Ce_0.25_Zr_0.75_O_2_ support during the reduction of the Pt­(IV)-rich layer in the calcination process of the as-prepared material.^55^ High-angle annular dark-field (HAADF) scanning transmission electron microscopy (STEM) images of the synthesized catalyst are shown in Figure 5a,b. Pt particles, exhibiting a relatively uniform size distribution, are clearly identified as brighter regions in the contrast, due to the higher atomic number of the element. In addition, the spectrum (Figure 5c) obtained from the selected area (Figure 5b) using energy-dispersive X-ray spectroscopy (EDX) reveals Pt peaks, including the Pt Lα peak at ∼9.4 keV. The powder X-ray diffraction pattern of the catalyst, along with Rietveld refinement results (Figure 5d and Table S5), indicates a tetragonal-phase Ce_0.25_Zr_0.75_O_2_ with a quantified Pt phase content of 4.1 wt %. This is consistent with the nominal Pt loading of 5 wt % to Ce_0.25_Zr_0.75_O_2_ during synthesis (equivalent to a Pt loading of 4.8 wt % in the catalyst).

**Figure 5:**
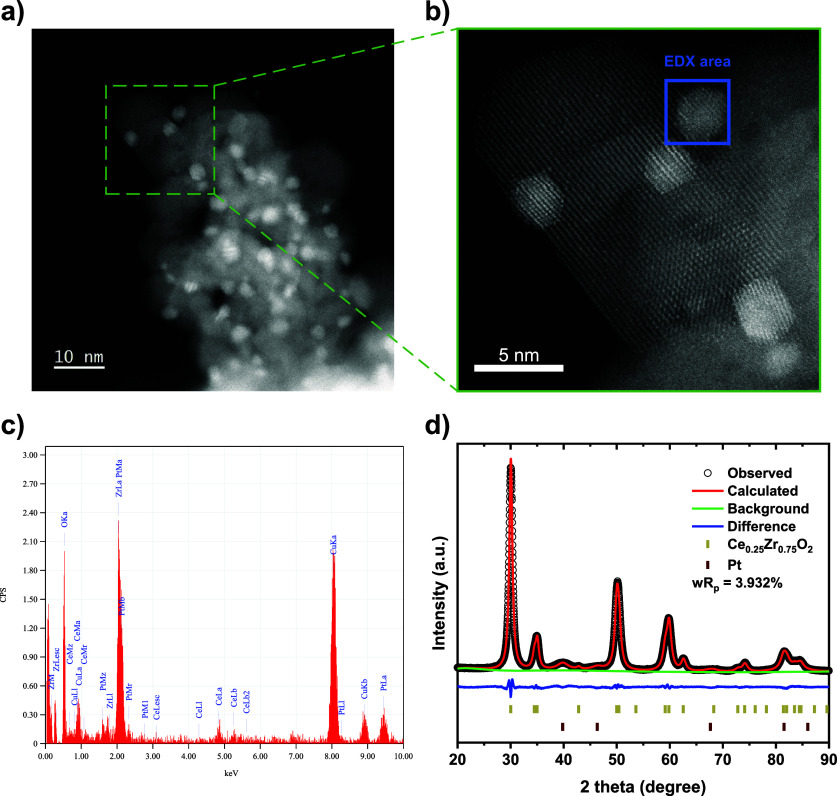
HAADF-STEM images of the synthesized Pt/Ce_0.25_Zr_0.75_O_2_ (a, b), with the EDX spectrum (c) acquired from the selected area in the image (Cu signals originate from the Cu grid used). The PXRD pattern with Rietveld refinement is presented in (d).

Substitution of Pt with a Pt/Ce_0.25_Zr_0.75_O_2_ interlayer near the anode in the composite membrane leads to the configuration *Ce*_0.25_ *Zr*_0.75_ *O*_2_ *(ca) - Pt/Ce*_0.25_ *Zr*_0.75_ *O*_2_ *(an)*, as illustrated in Figure 4c. This configuration exhibits the lowest fluoride release rate, measured at 0.023 (μg·cm^‑2^·h^‑1^) at the end of the 100-h constant current test (Figure 4d). Its average FRR of 0.028 ± 0.008 (μg·cm^–2^·h^–1^) represents a 3.6-fold reduction compared to *Ce*_0.25_ *Zr*_0.75_ *O*_2_ *(ca) - Pt (ca)* (0.10 ± 0.01 (μg·cm^–2^·h^–1^)). In the meantime, it also demonstrates the lowest H_2_ in O_2_% (Figure 4e), recorded at 1.6% at 0.5 A/cm^2^, corresponding to a 45% decrease relative to *Ce*_0.25_ *Zr*_0.75_ *O*_2_ *(ca) - Pt (an)*. Given the same Pt loading, this decrease in the recorded H_2_ in O_2_% may be attributed to the highly dispersed Pt nanoparticles on the Ce_0.25_Zr_0.75_O_2_ support in the Pt/Ce_0.25_Zr_0.75_O_2_ interlayer, providing more active surface area for efficient gas recombination, whereas they tend to remain agglomerated in the spray-coated Pt interlayer in *Ce*_0.25_ *Zr*_0.75_ *O*_2_ *(ca) - Pt (ca)*. Notably, compared to the 7.0% H_2_ in O_2_% measured in the blank membranes (Figure 2d) at 0.5 A/cm^2^, a 4.4-fold reduction is achieved when using the configuration *Ce*_0.25_ *Zr*_0.75_ *O*_2_ *(ca) - Pt/Ce*_0.25_ *Zr*_0.75_ *O*_2_ *(an)*.

The measured FRR allows us to estimate the lifetime of the membrane in the cell. For this, we assume that membrane end of life is marked by a loss of 10% of its fluoride inventory, based on an early mechanistic model for uniform PFSA membrane degradation.^13^ Although applicability to today’s state-of-the-art PFSA ionomers may be limited, we can nevertheless derive a useful trend, as membrane lifetime, if limited by chemical degradation, is expected to inversely scale with FRR.^56^ For Nafion NR 212 with a dry thickness of 50 μm, assuming a constant FRR of 0.023 (μg·cm^–2^·h^–1^) at a cell temperature of 90 °C, which was measured at the end of the 100-h constant current test, this yields a projected membrane lifetime of 29,000 h. Compared to blank measurements without any functional catalyst particle interlayers, this represents a 7.4 times increase. Using an apparent activation energy for membrane degradation of 58 kJ/mol,^13,57^ this translates to a lifetime of 50,000 h at 80 °C and 89,000 h at 70 °C. We neglect other degradation mechanisms and failure modes here, such as catalyst aging and mechanical degradation of the membrane, for example, caused by creep. Moreover, we assume that the activity of the ceria-zirconia radical scavenger is maintained over this time period. This certainly needs to be investigated further, as ceria is known to be thermodynamically unstable at low pH.^58^

Nevertheless, these results showcase the effectiveness of the synthesized Pt/Ce_0.25_Zr_0.75_O_2_ embedded as an interlayer in the composite membrane, in mitigating radical-induced ionomer degradation and hydrogen crossover. Based on the outcomes of the current study, the composite membrane *Ce*_0.25_ *Zr*_0.75_ *O*_2_ *(ca) - Pt/Ce*_0.25_ *Zr*_0.75_ *O*_2_ *(an)* demonstrates promising potential for application as next-generation catalyst-coated membranes in PEM water electrolysis cells.

Other future research can be conducted in terms of addressing the interfacial effect in the composite membrane that causes the increase in HFR. As shown in Figure 4f, the substitution with a thicker Pt/Ce_0.25_Zr_0.75_O_2_ interlayer compared to Pt results in an increased cell potential in the polarization curve, mainly due to a pronounced increase in HFR (Figure 4g). Electrochemical impedance spectra (EIS) for these samples at different current densities can be found in Figure S5. This could result from both an increased membrane thickness and enhanced interfacial effects. Alternative composite membrane fabrication methods beyond spray coating should be explored to improve interfacial properties. Additionally, optimizing the loadings of Ce_0.25_Zr_0.75_O_2_ and Pt/Ce_0.25_Zr_0.75_O_2_, as well as the interlayer ionomer-to-catalyst ratio, could help achieve an optimal balance between membrane electrical resistance and the functional performance of the interlayer.

## 3 Conclusions

In the current study, the effectiveness of functional interlayer positioning inside a composite membrane was investigated. Specifically, a radical scavenger interlayer, using Ce_0.5_Zr_0.5_O_2_ as a representative compound, was determined to be more effective when it is positioned near the cathode, due to its proximity to the location where reactive oxygen species are mostly expected to be generated. A reduction of 61% ± 7% in FRR was achieved in this configuration compared to its positioning near the anode. An optimization of Ce content in Ce_*x*_Zr_1*‑x*_O_2_ was conducted using an ex situ Fenton test, where Ce_0.25_Zr_0.75_O_2_ demonstrated the highest radical scavenging activity among the three compositions. Under the used cell testing conditions, a gas recombination catalyst interlayer, with Pt as an example, was confirmed to be more effective near the anode, showing a 1.7-fold reduction in H_2_ in O_2_% compared to its counterpart when positioned near the cathode. This is attributed to a closer match of the concentration of reactants with the stoichiometric ratio of the recombination reaction. Nevertheless, the Pt nanoparticles were replaced by a bi-functional catalyst Pt/Ce_0.25_Zr_0.75_O_2_ due to the Pt-induced additional ionomer degradation. A composite membrane configuration with a Ce_0.25_Zr_0.75_O_2_ interlayer positioned near the cathode and a Pt/Ce_0.25_Zr_0.75_O_2_ interlayer near the anode was proposed based on these findings and consequently examined. This configuration achieved an enhanced mitigation of both ionomer degradation and hydrogen crossover, yielding a 7.4-fold increase in the projected membrane lifetime at the testing conditions and a 4.4-fold decrease in H_2_ in O_2_% at 0.5 A/cm^2^ compared to blank membranes without any functional catalyst particle interlayers. The report highlights the importance in the rational design of composite membranes with functional interlayers for extended lifetimes and reduced gas crossover in PEM water electrolysis cells.

## 4 Experimental Section

### 4.1 Particle Synthesis

Powders of Ce_*x*_Zr_1‑*x*_O_2_ (*x* = 0.25, 0.5, and 0.75) and Pt/Ce_0.25_Zr_0.75_O_2_ were synthesized using a one-pot polyol method.^55,59^ Stoichiometric amounts of Ce­(NO_3_)_3_·6H_2_O (>99%, Sigma-Aldrich) and ZrOCl_2_·8H_2_O (98%, Sigma-Aldrich) were dissolved in ethylene glycol (EG, > 99.5%) to achieve a total metal ion concentration of 0.1 mol/L. A second solution was prepared by dissolving NaOH (>98%, Sigma-Aldrich) in an equal volume of EG with the addition of a small amount of Milli-Q water (3 vol % of the EG amount). The NaOH concentration in EG was maintained at 2.5 times the total metal ion concentration of the first solution. Both solutions were stirred, heated to 80 °C, and subsequently cooled to room temperature. The two solutions were then mixed, heated at 160 °C for 30 min with continuous stirring, and allowed to cool to room temperature again.

For Pt/Ce_0.25_Zr_0.75_O_2_, a third solution of H_2_PtCl_6_ with a Pt concentration of 9.05 mg_Pt_/mL was prepared and added to the mixture for a targeted 5 wt % Pt loading to Ce_0.25_Zr_0.75_O_2_. The resulting suspension was stirred and heated at 180 °C for 30 min, then cooled to room temperature.

The suspension was centrifuged and the resulting sediment was collected. The sediment was cleaned by sequentially washing it twice with ethanol and once with acetone. After each washing step, it was subject to centrifugation and redispersion via sonication. The obtained material was dried in an oven and ground into a fine powder with a mortar and pestle.

For Ce_*x*_Zr_1‑*x*_O_2_ (x = 0.25, 0.5, and 0.75), the corresponding powder was calcined in air at 500 °C for 3 h in order to achieve a relatively small crystallite size.

The calcination of Pt/Ce_0.25_Zr_0.75_O_2_ was conducted in 5% H_2_ with balanced He at 750 °C for 2 h in an alumina crucible, following conditions similar to one of those reported in the literature.^55^ The process was carried out in a thermogravimetric analyzer (TGA/DSC 1, Mettler Toledo), with the evolved gas analyzed via mass spectrometry to ensure accurate control of the reducing conditions.

### 4.2 Preparation of Composite Membranes with Interlayer Structure

The composite membrane was prepared through sequential spray-coating (ExactaCoat, Sonotek) of the targeted layers onto a Nafion NR 212 membrane base. All functional particle-containing layers were deposited using inks made by mixing the functional particle, water, isopropanol and ionomer dispersion (Nafion D520CS, Chemours) in a weight ratio of 1:5.2:1.3:20. During the spray-coating process, the membranes and their supporting frames were placed on a heated metal plate controlled at either 50 or 60 °C.

For the Ce_0.5_Zr_0.5_O_2_ layer, in-house synthesized Ce_0.5_Zr_0.5_O_2_ powder was used, with a sprayed Ce loading of 0.37 mg_Ce_/cm^2^ (equivalent to a Ce_0.5_Zr_0.5_O_2_ loading of 0.78 mg/cm^2^). An additional ionomer layer was applied on top of the Ce_0.5_Zr_0.5_O_2_ layer, by spraying only an ionomer dispersion (Nafion D521CS, Chemours) with a target dry ionomer loading of 0.05 mg/cm^2^ (corresponding to an approximate ionomer film thickness of 5 μm). Consequently, the composite membrane prepared with the Ce_0.5_Zr_0.5_O_2_ layer near the anode is denoted as *Ce*_0.5_ *Zr*_0.5_ *O*_2_ *(an)*, while the configuration with the Ce_0.5_Zr_0.5_O_2_ layer near the cathode is denoted as *Ce*_0.5_ *Zr*_0.5_ *O*_2_ *(ca)*.

The Ce_0.25_Zr_0.75_O_2_ layer was deposited similarly to the Ce_0.5_Zr_0.5_O_2_ layer, maintaining a constant Ce loading of 0.37 mg_Ce_/cm^2^ (equivalent to a Ce_0.25_Zr_0.75_O_2_ loading of 1.43 mg/cm^2^). This layer was specifically positioned near the cathode side of the Nafion NR 212 membrane. Then, (i) a Pt layer, where commercial Pt nanoparticles (HiSPEC 1000 Platinum Black, Johnson Matthey) were used, was applied either directly on the Ce_0.25_Zr_0.75_O_2_ layer near the cathode, denoted as *Ce*_0.25_ *Zr*_0.75_ *O*_2_ *(ca) - Pt (ca)*, or near the anode onto the Nafion NR212 membrane, denoted as *Ce*_0.25_ *Zr*_0.75_ *O*_2_ *(ca) - Pt (an)*. The target Pt loading was set as 0.04 mg_Pt_/cm^2^. In the other case, (ii) a Pt/Ce_0.25_Zr_0.75_O_2_ layer, instead of a Pt layer, was sprayed near the anode onto the Nafion NR 212 membrane and denoted as *Ce*_0.25_ *Zr*_0.75_ *O*_2_ *(ca) - Pt/Ce*_0.25_ *Zr*_0.75_ *O*_2_ *(an)*. The Pt/Ce_0.25_Zr_0.75_O_2_ catalyst was synthesized in-house with a Pt-to-Ce_0.25_Zr_0.75_O_2_ ratio of 5 wt %. During the spray coating, a Pt/Ce_0.25_Zr_0.75_O_2_ loading of 0.84 mg/cm^2^ (corresponding to a Ce loading of 0.21 mg_Ce_/cm^2^ and a Pt loading of 0.04 mg_Pt_/cm^2^) was achieved. In both scenarios, an additional ionomer layer (Nafion D521CS, Chemours; with a dry ionomer loading of 0.05 mg/cm^2^) was sprayed on top of the Pt or Pt/Ce_0.25_Zr_0.75_O_2_ layer.

### 4.3 Electrochemical Measurements

The catalyst-coated membrane was prepared by spray-coating the cathode and anode catalyst layers onto the composite membrane, as described previously.^29^ Pt/C catalyst (46.1 wt % Pt, TEC10E50E, Tanaka) was used at the cathode with a Pt loading of 0.4 mg_Pt_/cm^2^, and IrO_2_/TiO_2_ catalyst (74.1 wt % Ir, Elyst Ir75 0480, Umicore) was used at the anode with an Ir loading of 2 mg_Ir_/cm^2^. The CCM was assembled into a PEM water electrolysis cell with an active area of 25 cm^2^, using a carbon based gas diffusion layer (E20H, Freudenberg) on the cathode and a titanium-felt porous transport layer (2GDL20-1,00, Bekaert) on the anode,^29^ which was mounted onto a home-built electrolysis test bench. The configuration of this electrolysis test bench, including gas-water separators, gas flow and pressure controllers (Bronkhorst), a fluoropolymer inline heater (TIH3, Process Technology) and an ion exchange resin (BWT) integrated into the anode water loop, among the other components, has been described in detail previously.^60^ Electrochemical measurements were performed at 90 °C, with the cathode pressure set to 11 bar_a_ and the anode kept at ambient pressure. The freshly assembled cell was conditioned at 2 A/cm^2^ for 12 h prior to testing. Polarization curves were measured galvanostatically between 0.008 and 3 A/cm^2^, with a superimposed AC frequency of 10 kHz to determine high-frequency resistance. The H_2_ in O_2_% readings were recorded at various current density steps, with the final value at each step calculated as the average of the data from the last 5 min. Rate of ionomer degradation was evaluated in 100-h constant current test at 2 A/cm^2^. During the test, cathode effluent water samples were periodically collected by placing a Falcon tube at the outlet and operating the corresponding valves located on the downstream tubing connected to the cathode water-gas separator. The effluent water sample was analyzed to determine the fluoride concentration by ion chromatography (Metrohm 882 Compact IC plus). The fluoride release rate was calculated based on the fluoride concentration in the water sample and the flow rate of electro-osmotic drag water through the cell.

### 4.4 Characterizations

Powder X-ray diffraction was performed at room temperature in the 2θ range of 20°–90° with a step size of 0.02° (Bruker D8 Advance, Cu Kα radiation source). The obtained PXRD patterns were refined by the conventional Rietveld method using General Structure Analysis System - II (GSAS II) software.^61^

A scanning electron microscope (Zeiss NVision 40) was used to image the cross-section of the CCMs. Samples were prepared by cryo-fracturing, with the fractured pieces subsequently mounted onto the sample holders with carbon tape.

HAADF-STEM images of the synthesized Pt/Ce_0.25_Zr_0.75_O_2_ were acquired using a transmission electron microscope (JEOL JEM-ARM200F, 200 kV). Energy-dispersive X-ray spectrum on the selected sample area was measured for elemental composition analysis.

Nitrogen physisorption was conducted using a QuantaChrome Autosorb IQ. Approximately 100 mg of each sample was loaded into a 6 mm borosilicate glass adsorption tube. The samples were degassed under vacuum at 250 °C for 8 h prior to analysis to remove adsorbed water and contaminants. Surface area quantification was performed using multipoint BET analysis in a relative pressure (*p*/*p*_0_) range of 0.1 to 0.3 of the adsorption data points.^62^ The BJH method was used on desorption data to determine pore size distribution and cumulative pore volume.^63^

Fluoride concentration in the samples taken from ex situ Fenton test solution was quantified using an ion chromatography instrument (Metrohm 882 Compact IC plus).

### 4.5 Ex Situ Fenton Test

A Nafion NR 212 membrane, cut into the dimensions of 22 mm × 22 mm, was placed between two PTFE meshes and positioned in a 25 mL glass vial together with a magnetic stirring bar. To this setup, 10 mL of hydrogen peroxide (H_2_O_2_, > 30% w/v, Fisher Scientific) solution and 10 mL of 40 mg/L iron sulfate (FeSO_4_·7H_2_O, Merck) solution were freshly mixed and added. The pH of the mixture was adjusted by introducing 0.02 mL of 1 M trifluoroacetic acid. Three in-house synthesized cerium-zirconium oxide samples with varying Ce/Zr ratios (Ce_*x*_Zr_1‑*x*_O_2_, *x* = 0.25, 0.5 and 0.75) were individually added to the separate vials, ensuring a consistent Ce mass across all samples. Specifically, 9.7 mg of Ce_0.25_Zr_0.75_O_2_, 5.3 mg of Ce_0.5_Zr_0.5_O_2_ and 3.8 mg of Ce_0.75_Zr_0.25_O_2_ were used in the respective experiments. The vials were placed on a stirring plate and stirred continuously at room temperature for the duration of the experiments. For each Ce/Zr ratio, three parallel experiments were carried out. At a defined time interval (22, 46, and 70 h from the start of the experiment), a 2 mL sample was taken from the reaction mixture, centrifuged, and the supernatant was collected. The fluoride concentration in the supernatant was analyzed using ion chromatography to monitor the chemical degradation of the membrane.

## Supplementary Material



## Notes

Supporting Information: electro-osmotic drag water rates, determined fluoride release rates, electrochemical performance of the tested membranes, nitrogen physisorption analysis, Ce 3d XPS results of Ce_*x*_Zr_1‑*x*_O_2_ (*x* = 0.25, 0.5 and 0.75), EIS spectra, and structural parameters obtained from Rietveld refinement of the PXRD pattern for Pt/Ce_0.25_Zr_0.75_O_2_ (PDF)

## #

Institute of Biological and Chemical Systems—Functional Molecular Systems, Campus North, Karlsruhe Institute of Technology, 76344 Eggenstein-Leopoldshafen, Germany
