# Supplementary material for: Composite Proton Exchange Membranes with Interlayer Structure Containing Functional Catalyst Particles for Water Electrolysis
Source: ACS Appl Mater Interfaces. 2025 Sep 18;17(39):54656–67. doi: 10.1021/acsami.5c08461 (PMC12492325; doi:10.1021/acsami.5c08461)
Supplement: Supplementary file 1 [file am5c08461_si_001.pdf]

Supporting Information: Composite Proton Exchange Membranes with Interlayer Structure Containing Functional Catalyst Particles for Water Electrolysis

Zheyu Zhang[1], Masis Sirim[1,†], Dominika Baster[1], Mario El Kazzi[1], Andrea Testino[1,2] and Lorenz Gubler[1,]*

[1] PSI Center for Energy and Environmental Sciences, Villigen PSI 5232, Switzerland

[2] STI SMX-GE, École Polytechnique Fédérale de Lausanne, Lausanne 1015, Switzerland

* Corresponding Author: lorenz.gubler@psi.ch

Present Address

[†] Institute of Biological and Chemical Systems - Functional Molecular Systems, Campus North, Karlsruhe Institute of Technology, 76344 Eggenstein-Leopoldshafen, Germany

Table S1. Electro-osmotic drag rates of water measured during 100-hour constant current measurements at 2 A/cm^2 (90 °C, p_c = 11 bar, p_a = 1 bar). The calculation steps can be found in earlier work.[1]

Composite membrane configuration	Electro-osmotic drag rate of water (mL·h^{-1}·cm^{-2})	Electro-osmotic drag coefficient
$Ce_{0.5}Zr_{0.5}O_2$ (an)	6.0	4.5
$Ce_{0.5}Zr_{0.5}O_2$ (ca)	5.8	4.3
$Ce_{0.25}Zr_{0.75}O_2$ (ca) – Pt (an)	5.5	4.1
$Ce_{0.25}Zr_{0.75}O_2$ (ca) – Pt (ca)	5.5	4.1
$Ce_{0.25}Zr_{0.75}O_2$ (ca) – Pt/$Ce_{0.25}Zr_{0.75}O_2$ (an)	5.6	4.2

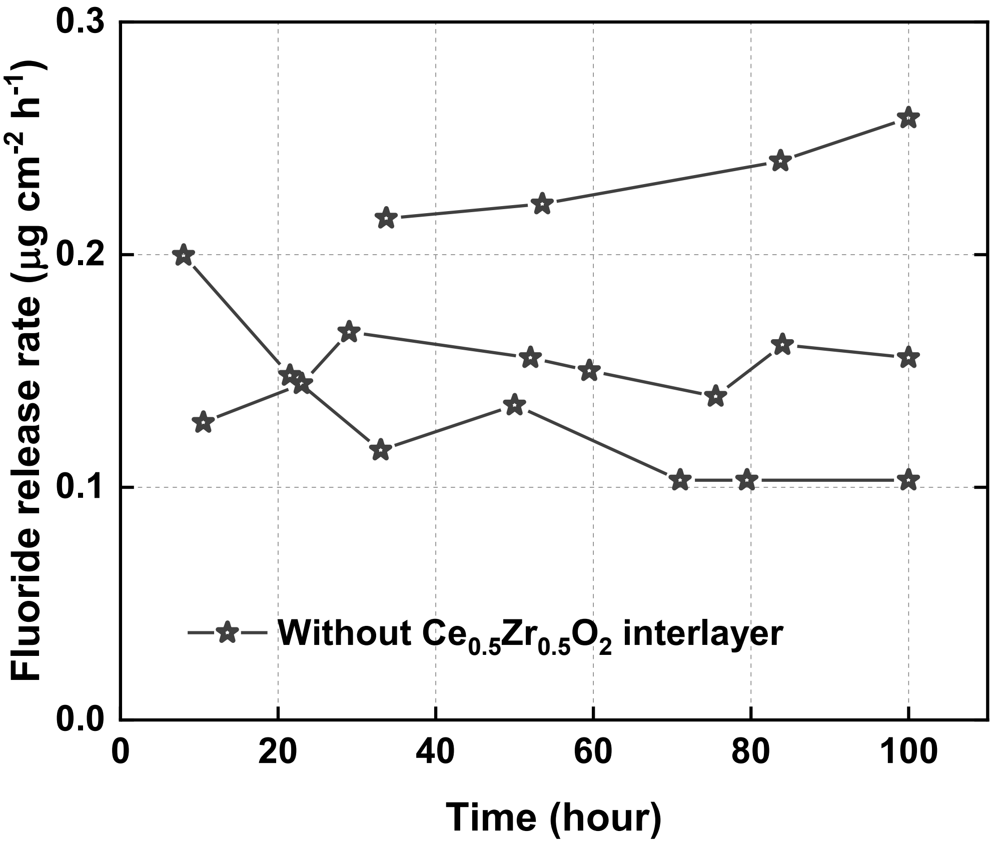

Figure S1. Fluoride release rates (FRRs) from three individual blank measurements without any $Ce_{0.5}Zr_{0.5}O_2$ interlayer determined during 100-hour constant current tests. For each test, the FRRs over the 100-hour period were first averaged to obtain the mean and standard deviation for that individual measurement. Subsequently, the overall average and standard deviation across the three tests were calculated with appropriate error propagation, resulting in an average FRR of 0.17 ± 0.01 ($\mu g \cdot cm^{-2} \cdot h^{-1}$), as described in the manuscript.

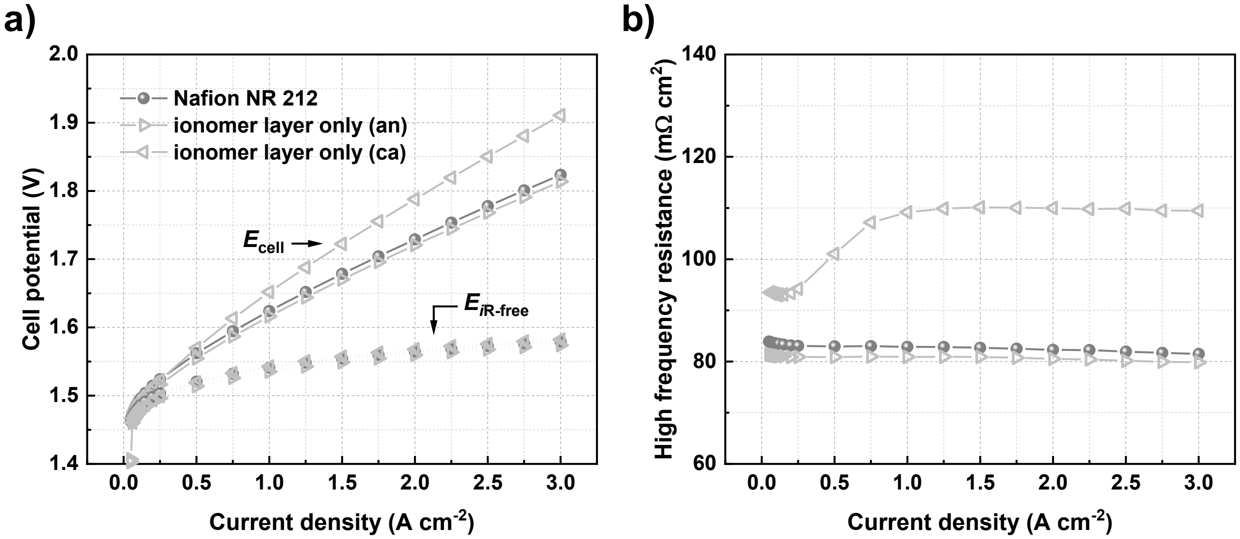

Figure S2. Polarization curves and *i*R-free potentials using a Nafion NR 212 membrane, a composite membrane with an ionomer layer added near the anode, and a composite membrane with an ionomer layer added near the cathode (a). The corresponding measured high-frequency resistances are shown in (b).

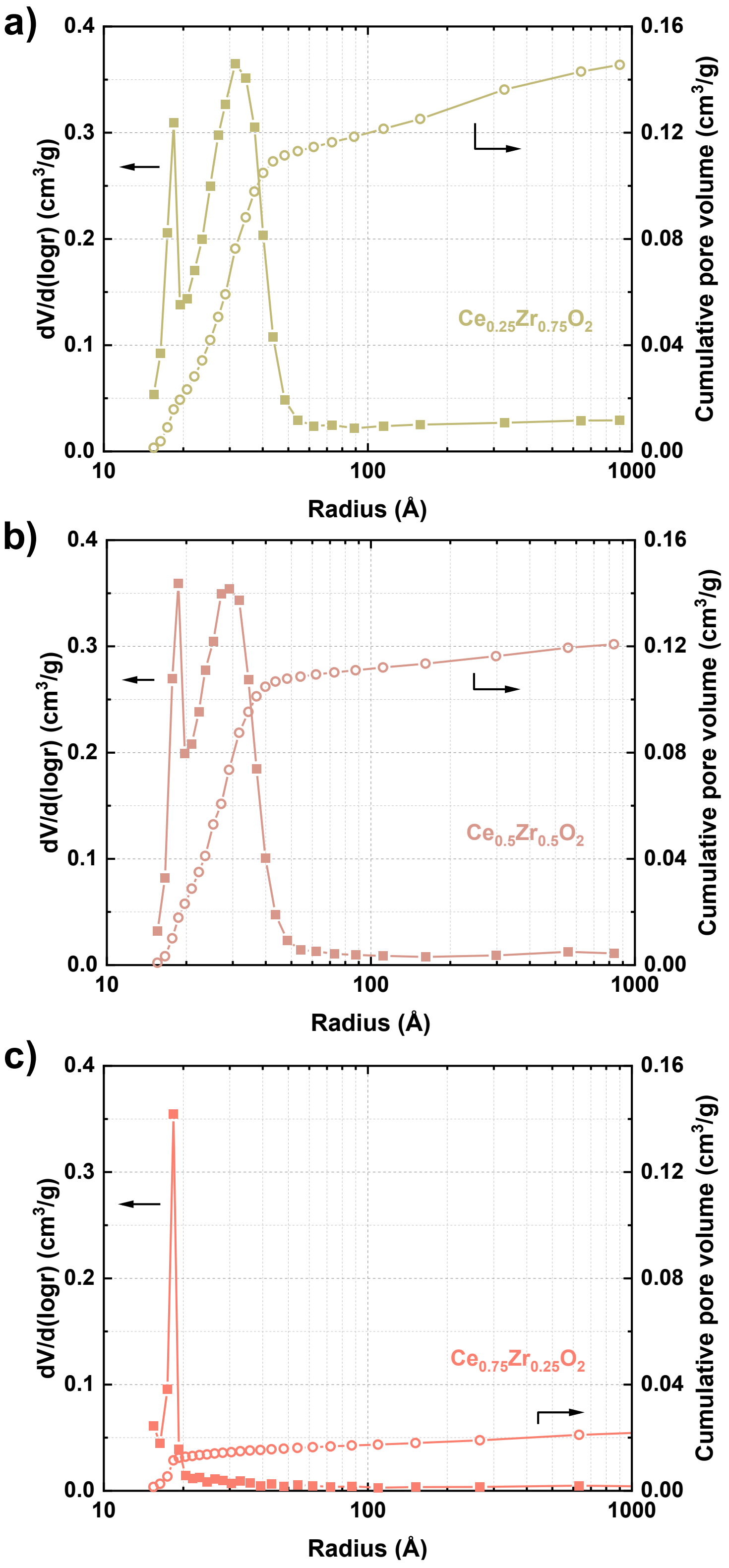

Figure S3. Pore size distribution and cumulative pore volume determined by the BJH method using N_2 desorption isotherm for $Ce_{0.25}Zr_{0.75}O_2$ (a), $Ce_{0.5}Zr_{0.5}O_2$ (b), and $Ce_{0.75}Zr_{0.25}O_2$ (c).

Table S2. Comparison of the crystallite size determined by the BET surface area in this study and that obtained by Rietveld refinement in the referenced work.

Sample	Crystallite size (nm) determined by the BET surface area*	Crystallite size (nm) determined by Rietveld refinement[2]
$Ce_{0.25}Zr_{0.75}O_2$	13.2	13.2
$Ce_{0.5}Zr_{0.5}O_2$	15.3	13.5
$Ce_{0.75}Zr_{0.25}O_2$	13.1	9.9

* The BET equivalent diameter (d_{BET}) for crystallite size estimation was calculated, based on the sample density (ρ_{XRD}) determined by XRD and the specific surface area (SSA) determined by the BET method, using the equation:

$$d_{BET} = \frac{6}{\rho_{XRD} \cdot SSA}$$

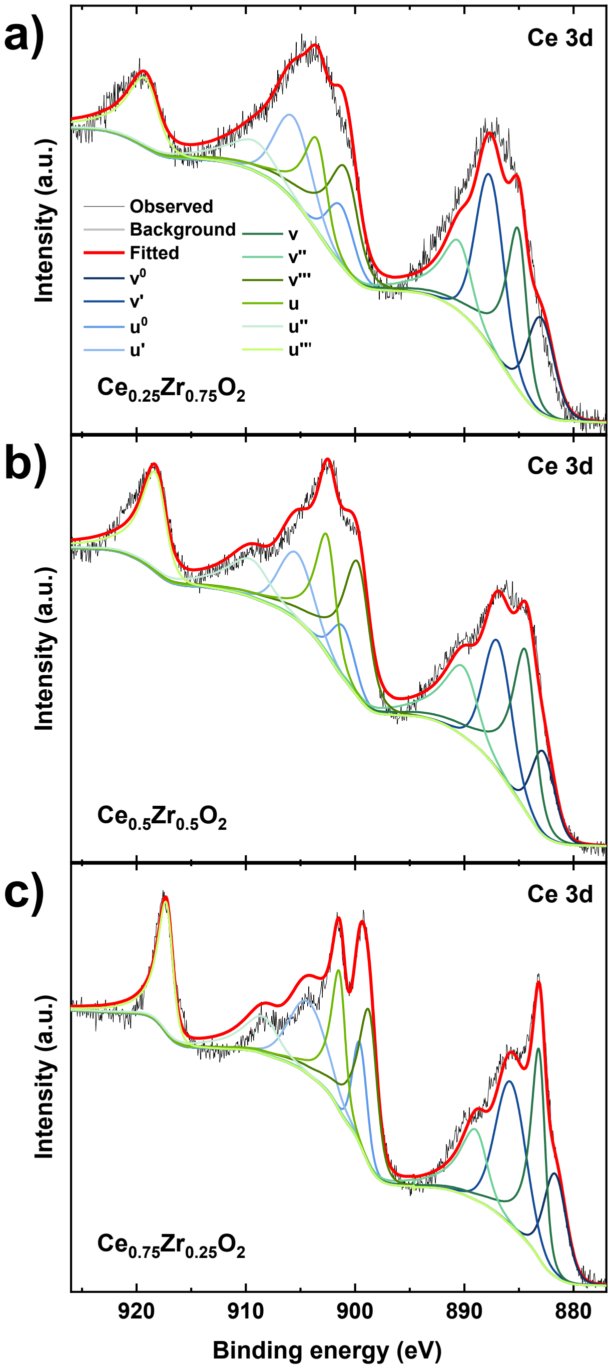

Figure S4. Ce 3d XPS results of $Ce_{0.25}Zr_{0.75}O_2$ (a), $Ce_{0.5}Zr_{0.5}O_2$ (b), $Ce_{0.75}Zr_{0.25}O_2$ (c) powders. The experimental data are shown as black curves and fit to the data including a sum of the peak fits as colored curves.

The X-ray Photoelectron Spectroscopy (XPS) analysis was performed with a VG ESCALAB 220iXL spectrometer (Thermo Fisher Scientific) using focused monochromatized Al Kα radiation (1486.6 eV) with a beam size of ~500 μm^2. The analysis chamber had a recorded residual pressure of approximately 2×10^{-9} mbar. The spectrometer was regularly calibrated using the Ag 3d5/2 peak with a binding energy of 368.3 eV with a full width at half maximum (FWHM) of 0.78 eV at a pass energy of 30 eV. All survey spectra were recorded with a dwell time of 50 ms, using the pass energy of 70 eV in steps of 0.5 eV. The spectra acquired in a narrow energy scan were recorded with the pass energy of 20 eV in a step size of 0.05 eV. Measurements were performed directly on the powder samples grounded in the agate mortar with 10 wt% of carbon black (C-NERGY™ SUPER C65T, Imerys S.A.).

All spectra were calibrated relative to the carbon C 1s peak at 284.8 eV to correct for charging effects. Curve fitting of the core level spectra was performed with the CasaXPS software. Background removal was achieved using the Shirley background. The peak fitting was performed using the generally accepted approach to fit the spectra with a complex peak model consisting of 10 peaks in total.[3,4] The concentration of Ce (IV) and Ce (III) can be found based on the relative percentages of each set of peaks. The peaks in Ce^{4+} 3d analysis are typically labeled u/v (Ce $3d^9 4f^2$ O $2p^4$ final state), u^{II}/v^{II} (Ce $3d^9 4f^1$ O $2p^5$ final state) and u^{III}/v^{III} (Ce $3d^9 4f^0$ O $2p^6$ final state).[5] The peaks in Ce^{3+} 3d analysis are typically labeled u^0/v^0 (Ce $3d^9 4f^2$ O $2p^5$ final state) and u^I/v^I (Ce $3d^9 4f^1$ O $2p^6$ final state).[5] The analysis of Ce 3d XPS spectra was carried out based on results published by Romeo *et al.*[3] and D.J. Morgan[4]. The Ce (III) components were fitted using a Voigt-like function, defined within the software by LA (1.53,243), while the more asymmetric-like components of the Ce (IV) spectra were fitted using LA (0.9,2,50).[5] The results are given in Tables S3 and S4.

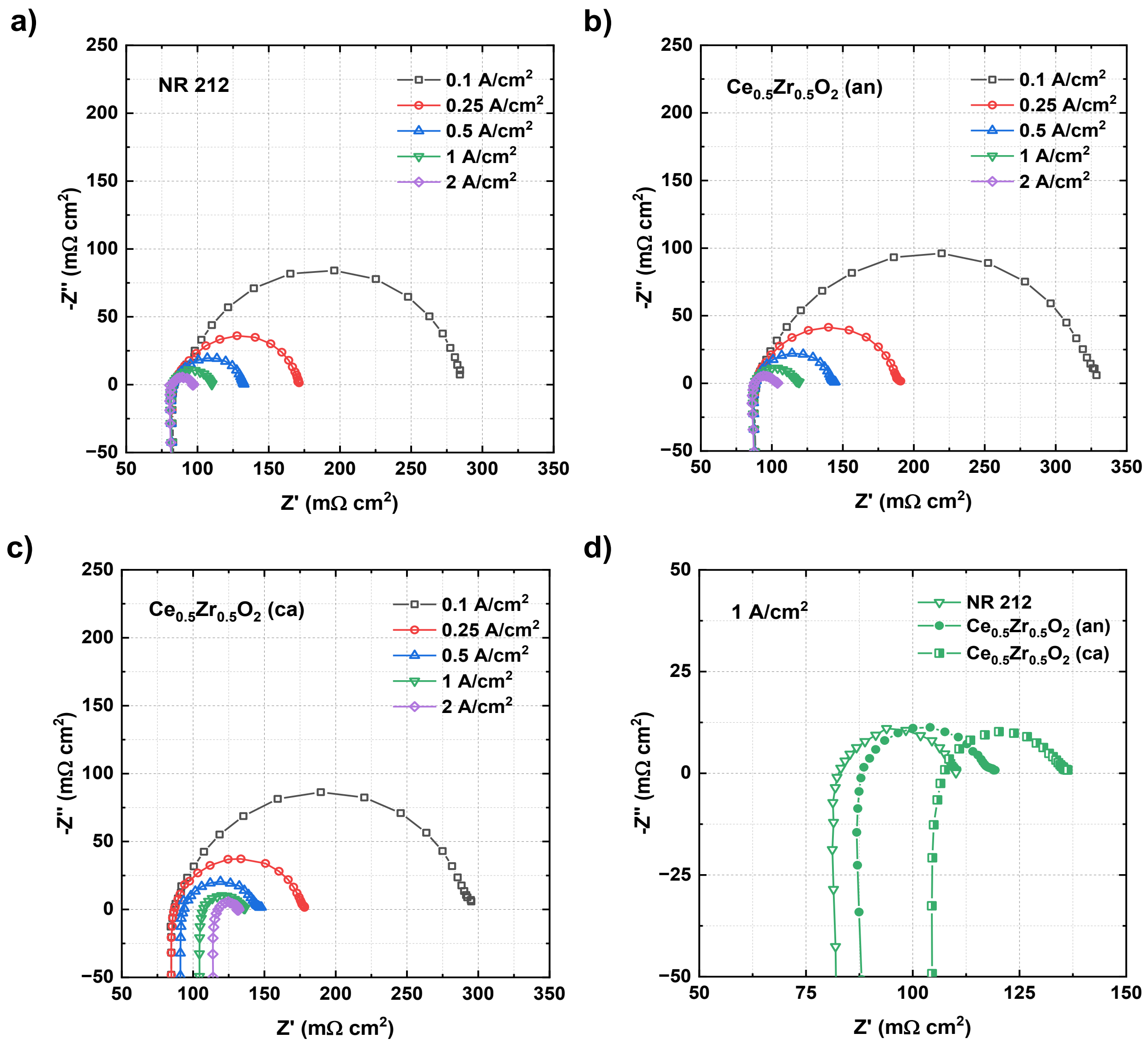

Figure S5. Measured EIS spectra for the Nafion NR 212 reference (a), *$Ce_{0.5}Zr_{0.5}O_2$ (an)* (b), and *$Ce_{0.5}Zr_{0.5}O_2$ (ca)* (c) in the current density range of 0.1–2 A/cm^2, EIS spectra of the 3 samples at 1 A/cm^2 (d).

EIS spectra for the Nafion NR 212 reference, *$Ce_{0.5}Zr_{0.5}O_2$ (an)*, and *$Ce_{0.5}Zr_{0.5}O_2$ (ca)* are shown in Figure S5. It can be seen that the x-axis intercept of the EIS spectra – corresponding to the ohmic resistance – remains relatively stable for both the NR 212 reference and the *$Ce_{0.5}Zr_{0.5}O_2$ (an)* over the measured current density range (0.1–2 A/cm^2). In contrast, for *$Ce_{0.5}Zr_{0.5}O_2$ (ca)*, the x-axis

intercept increases with increasing current density starting from 0.25 A/cm^2, consistent with the trend observed in the HFR data presented in Figure 2c. The x-axis intercept in all EIS spectra, corresponding to the value of Z” closest to zero, occurs at a frequency of 880 Hz or 1.3 kHz.

It is generally understood that three main sources accounts for the measured HFR: 1) membrane resistance, 2) electrical resistance of cell components, and 3) interfacial/contact resistance. Under the current experimental conditions, the membrane is assumed to be fully hydrated, and the electrolysis cell and its components remained unchanged. Therefore, the membrane and electrical resistance are expected to remain constant. Thus, we attribute the observed increase in HFR for $Ce_{0.5}Zr_{0.5}O_2$ *(ca)* to the interfacial effects, arising from the presence of the additional sprayed interlayer near the cathode, as discussed in the relevant sections of the manuscript.

Table S3. Experimental binding energies and full width at half-maximum (γ) of Ce 3d peaks.

x in $Ce_xZr_{1-x}O_2$	Ce^{3+}							
	**v^0**	γ	**v′**	γ	**u^0**	γ	**u′**	γ
**0.25**	883.00	2.90	887.66	3.02	901.30	2.90	905.65	3.95
**0.5**	882.78	2.50	886.94	3.01	901.08	2.50	905.38	3.67
**0.75**	881.69	2.49	885.75	3.27	899.56	1.54	904.29	4.07

	Ce^{4+}											
	**v**	γ	**v″**	γ	**v‴**	γ	**u**	γ	**u″**	γ	**u‴**	γ
**0.25**	885.00	1.90	890.40	2.79	900.80	2.90	903.40	2.2	909.00	4.37	919.10	2.94
**0.5**	884.31	2.14	889.99	3.19	899.65	2.50	902.50	1.93	909.39	3.97	918.20	2.31
**0.75**	883.11	1.34	888.88	2.55	898.64	1.76	901.39	1.34	908.28	3.27	917.23	1.43

Table S4. Ce^{3+} and Ce^{4+} concentrations determined by XPS analysis.

Sample	Ce^{3+} (%)	Ce^{4+} (%)
$Ce_{0.25}Zr_{0.75}O_2$	38	62
$Ce_{0.5}Zr_{0.5}O_2$	29	71
$Ce_{0.75}Zr_{0.25}O_2$	38	62

Table S5. Structural parameters obtained by Rietveld refinement of the PXRD pattern for $Pt/Ce_{0.25}Zr_{0.75}O_2$.

Phase	$Ce_{0.25}Zr_{0.75}O_2$			Pt
a (Å)	3.6107 ± 0.0002			3.907 ± 0.001
c (Å)	5.1835 ± 0.0003			-
V (Å^3)	67.58 ± 0.01			59.64 ± 0.05
Phase fraction (wt%)	95.9			4.1
Crystallite size (nm)	20.2			3.8
Microstrain	11973 (Equatorial)			2262
	16550 (Axial)			
Position	Ce	Zr	O	Pt
x	0.75	0.75	0.25	0
y	0.25	0.25	0.25	0
z	0.25	0.25	0.4545	0
Occupancy	0.25	0.75	1	1
U_{iso}	0.0426	0.0108	0.0158	0.004

References:

(1) Zhang, Z.; Han, Z.; Testino, A.; Gubler, L. Platinum and Cerium-Zirconium Oxide Co-Doped Membrane for Mitigated H_2 Crossover and Ionomer Degradation in PEWE. *J. Electrochem. Soc.* **2022**, *169* (10), 104501. https://doi.org/10.1149/1945-7111/ac94a3.

(2) Zhang, Z.; Pilger, F.; Alxneit, I.; Carino, A.; Tarik, M.; Müller, E.; Cervellino, A.; Mühlmann, A.; Ludwig, C.; Gubler, L.; Testino, A. Pt/$Ce_xZr_{1-x}O_2$ Bi-Functional Catalyst for Gas Recombination and Radical Scavenging in PEM Water Electrolysis Cells. *ACS Catal.* **2025**, *15* (7), 5577–5588. https://doi.org/10.1021/acscatal.4c07426.

(3) Romeo, M.; Bak, K.; El Fallah, J.; Le Normand, F.; Hilaire, L. XPS Study of the Reduction of Cerium Dioxide. *Surf. Interface Anal.* **1993**, *20* (6), 508–512. https://doi.org/10.1002/sia.740200604.

(4) Morgan, D. J. Photoelectron Spectroscopy of Ceria: Reduction, Quantification and the Myth of the Vacancy Peak in XPS Analysis. *Surf. Interface Anal.* **2023**, *55* (11), 845–850. https://doi.org/10.1002/sia.7254.

(5) Bêche, E.; Charvin, P.; Perarnau, D.; Abanades, S.; Flamant, G. Ce 3d XPS Investigation of Cerium Oxides and Mixed Cerium Oxide ($Ce_xTi_yO_Z$). *Surf. Interface Anal.* **2008**, *40* (3–4), 264–267. https://doi.org/10.1002/sia.2686.
